# O.1.1-5 Physical activity interventions and depressive symptoms in obese adults. An ongoing systematic review and meta-analysis

**DOI:** 10.1093/eurpub/ckad133.081

**Published:** 2023-09-11

**Authors:** Ioannis D Morres, Antonis Hatzigeorgiadis, Charalampos Krommidas, Nikos Comoutos, Odysseas Androutsos, Yannis Theodorakis

**Affiliations:** University of Thessaly, School of Physical Education, Sport Science and Dietetics, Trikala, Greece; iomorres@uth.gr; University of Thessaly, School of Physical Education, Sport Science and Dietetics, Trikala, Greece; iomorres@uth.gr; University of Thessaly, School of Physical Education, Sport Science and Dietetics, Trikala, Greece; iomorres@uth.gr; University of Thessaly, School of Physical Education, Sport Science and Dietetics, Trikala, Greece; iomorres@uth.gr; University of Thessaly, School of Physical Education, Sport Science and Dietetics, Trikala, Greece; iomorres@uth.gr; University of Thessaly, School of Physical Education, Sport Science and Dietetics, Trikala, Greece; iomorres@uth.gr

## Abstract

**Introduction:**

Physical activity (PA) ameliorates depressive symptoms in adults (Morres et al., 2019; Morres et al., 2022). However, meta-analytic evidence for the antidepressant effects of PA in obese adults in particular is equivocal but confounded by trials often examining various behavioral weight loss interventions, mixed overweight/obese samples, and non-depression specific outcomes. Therefore, this systematic review and meta-analysis synthesized evidence for the effects of exclusively PA interventions on depression outcomes in obese adults aged 18-65 years.

**Methods:**

Nine e-databases were searched for relevant randomized controlled trials published through January 31, 2023. Data were coded based on the PICOS criteria referring to Participants, Interventions, Comparisons, Outcomes and Study design. The Comprehensive Meta-Analysis software (CMA) computed a random-effect model (Hedge’s g criterion) to calculate pooled standardized mean differences for post-intervention depression scores with higher depression scores indicating more severe symptoms. The I^2^ and Cochran Q measured heterogeneity levels. Publication bias was investigated with the funnel plot visual inspection, the Begg-Mazumbar Kendall’s tau and Egger tests. Risk of bias analysis evaluated the methodological quality of the reviewed trials with the Physiotherapy Evidence Database Scale, which is designed for physical therapy interventions such as PA.

**Results:**

A total of 7 randomized controlled trials (N = 732 participants) met review criteria. PA interventions documented a statistically significant large overall antidepressant effect with high and statistically significant heterogeneity (g = -0.94, 95% Confidence Interval [CI] = -1.81, -0.54, p < 0.001; I^2^ = 87%, Cochran Q = 11.32, p < 0.05). The fail-safe criterion and the Mazumbar Kendall’s tau and Egger tests recorded publication bias. Trim-fill analysis on both sides of the plot added one study on the left side plot and adjusted-(increased) the effect accordingly (g = -1.12, 95% CI = -1.94, -0.85, p < 0.001). Risk of bias analysis showed an overall moderate scoring for trials’ methodological quality.

**Conclusions:**

Based on our preliminary findings, PA in obese adults brings about large antidepressant effects with high hetereogeneity, stressing the need for meta-regression analysis. Practitioners and policy-makers may consider that PA appears to be a promising antidepressant strategy for obese adults. However, well-designed trials are needed for firmer conclusions.

